# Plasma random glucose levels at hospital admission predicting worse outcomes in STEMI patients undergoing PCI: A case series

**DOI:** 10.1016/j.amsu.2022.103857

**Published:** 2022-05-29

**Authors:** Tooba Ahmed Kirmani, Manjeet Singh, Sumeet Kumar, Karan Kumar, Om Parkash, Sagar, Farah Yasmin, Farmanullah Khan, Najeebullah Chughtai, Muhammad Sohaib Asghar

**Affiliations:** a Dow University of Health Sciences, Karachi, Pakistan; b Liaquat National Hospital and Medical College, Karachi, Pakistan; c Ghulam Muhammad Mahar Medical College, Sukkur, Pakistan; d Chandka Medical College, Larkana, Pakistan; e Jinnah Sindh Medical University, Karachi, Pakistan

**Keywords:** Diabetes, Random blood sugar, Mortality, Cardiovascular outcomes, Case series

## Abstract

**Background:**

The effects of impaired plasma glucose levels on predicting clinical outcomes and in-hospital events in patients with ST-segment elevation myocardial infarction (STEMI) undergoing percutaneous coronary intervention (PCI) is unknown. Therefore, we evaluated random blood glucose at admission and its association with clinical outcomes in STEMI patients treated with PCI.

**Methods:**

Patients with STEMI undergoing PCI were enrolled and were divided into 4 tertiles according to random blood glucose levels. Tertile 1 had levels below 100 mg/dL, tertile 2 had 100–200 mg/dL, tertile 3 had 200–300 mg/dL, and tertile 4 had random blood glucose levels >300 mg/dL. The cumulative rates of in-hospital mortality and major adverse cardiovascular events were calculated.

**Results:**

Both the incidence of all-cause deaths and cumulative rates of major adverse cardiovascular events were significantly the lowest in patients within tertile 1. The cumulative incidence of in-hospital events was 14.3% in tertile 1, 17.6% in tertile 2, 23.5% in tertile 3, and 30.8% in tertile 4. The odds ratio of major adverse cardiovascular events was 1.286 [0.397–4.161] in tertile 2, 1.846 [0.492–6.927] in tertile 3, and 2.667 [0.693–10.254] in tertile 4. The cumulative proportion of adverse events was seen higher in tertile 4 on Kaplan-Meier log-rank (chi-square: 8.094, p = 0.044).

**Conclusion:**

Poor glycemic control or stress hyperglycemia on admission experienced the highest rates of major adverse cardiovascular events including deaths. Plasma random glucose was predictive of a worse prognosis for STEMI patients undergoing PCI in our study.

## Highlights

• Poor glycemic control or stress hyperglycemia on admission is considered to have a negative impact on acute cardiac events.
• Both the incidence of all-cause deaths and cumulative rates of major adverse cardiovascular events were significantly higher in patients with higher tertiles of random blood glucose levels.
• Plasma random glucose was predictive of a worse prognosis for STEMI patients undergoing PCI in our study.

## 1 Introduction

Ischemic heart disease puts global health significantly at risk and is an important cause of increasing mortality all around the world [1]. Notably, in patients with ST-segment elevation myocardial infarction (STEMI), the treatment with percutaneous coronary intervention (PCI) is recommended for timely restoration of coronary blood flow and reduced infarct size [2]. However, the risk of additional myocardial damage due to reperfusion of the affected myocardium remains [2]. Though the pathophysiology of reperfusion injury is fully not understood; hyperglycemia is a common finding in STEMI patients with an occurrence of up to 50% [3]. Furthermore, hyperglycemia has also been linked to reperfusion injury [2]. According to previous studies, hyperglycemia imposes a higher mortality risk and complications during in-hospital stay [4,5], including larger myocardial infarct size and major adverse cardiovascular events (MACE) [1,3,5]. Diabetes mellitus (DM) is a key factor in causing several cardiovascular complications but patients with and without known diabetes mellitus, both are affected by hyperglycemia [3,4]. Currently, acute hyperglycemic state is labeled as ≥180 mg/dL at the time of admission [5].

The current study aims to predict clinical course, characteristics, complications, and outcomes in STEMI patients undergoing PCI in relation to random blood glucose levels at admission.

## 2 Material and methods

This case series analyzed data from 190 Acute Myocardial Infarction patients hospitalized in the cardiac care unit of Dow University Hospital – Ojha Campus, Karachi, between March and October 2020. The diagnostic criteria for ACS were based on chest pain or discomfort, electrocardiogram (ECG) changes, and measurements of myocardial injury biomarkers such as Troponin I and Creatine kinase-MB (CK-MB). In-hospital events (or death) were the primary end outcome. Patient chart review and electronic medical records were accessed for data collection after ethical review board approval. The study included all the patients diagnosed with STEMI and undergoing primary PCI during their hospital stay. The procedure was performed by an interventional cardiologist with relevant training in the field for at least 4 years. Patient optimization prior to PCI, including anticoagulation and antiplatelet therapy was performed according to predefined in-hospital protocol. NSTEMI patients, those conservatively managed, having incomplete data, or random blood sugar levels not available at admission were excluded from the study. The patients were followed only for their in-hospital stay and no post-discharge outcomes were reported.

Patients were divided into 4 tertiles according to random blood glucose levels. Tertile 1 had levels below 100 mg/dL, tertile 2 had 100–200 mg/dL, tertile 3 had 200–300 mg/dL, and tertile 4 had random blood glucose levels >300 mg/dL.

The cumulative rates of in-hospital mortality and major adverse cardiovascular events were calculated. In-hospital outcomes included exacerbation of congestive heart failure (CHF) during hospitalization, severe arrhythmia, bleeding complication, new-onset or recurrent myocardial infarction (MI), stroke, and cardiogenic shock. Socio-demographic and clinical variables, Socio-demographic factors, and angiographic profiles were obtained from a retrospective chart review of all the patients, including age, gender, height, weight, and smoking status of the study participants. Clinical variables included comorbidities, history of previous cardiac events, presenting symptoms, Killip class, and infarct localization on ECG. The angiographic profile included vessel involvement, severity of coronary artery disease (CAD), post-operative thrombolysis in myocardial infarction (TIMI) score, stent diameter, and severity of lesion on angiography.

The study was prepared according to guidelines reported by STROCSS group [6]. The study protocol was registered with Dow University Hospital's registry (UIN# IRB/DUH/2020/784). Consent to participate was not required due to retrospective nature of the study. Data were analyzed using SPSS version 25.0 (IBM Corp. Armonk, NY, USA). Descriptive statistics were presented either as mean and standard deviation or frequency and percentages. Data was stratified for effect modification by using the tertiles of random blood glucose levels. Receiver operating characteristic analysis was performed to obtain the area under the curve (AUC) for random blood glucose predicting in-hospital events. Kaplan-Meier log-rank (Mantel-Cox) test was applied to signify in-hospital adverse events according to the tertiles of random blood glucose levels.

## 3 Results

The mean age of the cohort was 56.42 ± 11.74 years, and the mean body mass index (BMI) was 27.78 ± 5.01 kg/m^2^. Of the 190 included participants, 60% were males (n = 114), 27.3% had a prior history of MI, 23.7% had a prior history of PCI, and 5.3% had coronary artery bypass grafting (CABG). About 56.8% were known diabetics, 71% were hypertensive, and 11.6% had renal insufficiency (Table 1). A total of 94 patients presented with typical chest pain, and most (n = 96) were having pre-operative Killip class 2. Left anterior descending (LAD) was the most frequent infarct-related artery, and three-vessel disease was predominantly documented.

**Table 1:** Sociodemographic and clinical factors of study population (n = 190).

Socio-demographic and clinical variables	**Tertile 1 (<100 mg/dL) n = 28**	**Tertile 2 (101–200 mg/dL) n = 102**	**Tertile 3 (201**–**300 mg/dL) n = 34**	**Tertile 4 (>300 mg/dL) n = 26**
**Males (n = 114)**	20 (17.5%)	52 (45.6%)	26 (22.8%)	16 (14.0%)
**Smoker (n = 35)**	8 (22.6%)	18 (51.4%)	4 (11.4%)	5 (14.3%)
**Peripheral artery disease (n = 9)**	2 (22.2%)	2 (22.2%)	1 (11.1%)	4 (44.4%)
**Dyslipidemia (n = 42)**	6 (14.3%)	24 (57.1%)	8 (19.0%)	4 (9.5%)
**Hypertension (n = 135)**	19 (14.1%)	69 (51.1%)	26 (19.3%)	21 (15.5%)
**Diabetes mellitus (n = 108)**	6 (5.6%)	48 (44.4%)	30 (27.8%)	24 (22.2%)
**Atrial fibrillation (n = 10)**	1 (10.0%)	5 (50.0%)	2 (20.0%)	2 (20.0%)
**History of Stroke (n = 8)**	2 (25.0%)	4 (50.0%)	1 (12.5%)	1 (12.5%)
**Renal insufficiency (n = 22)**	0 (0.0%)	14 (63.6%)	6 (27.3%)	2 (9.1%)
**On hemodialysis (n = 6)**	0 (0.0%)	4 (66.7%)	2 (33.3%)	0 (0.0%)
**History of cardiac arrest (n = 2)**	0 (0.0%)	2 (100.0%)	0 (0.0%)	0 (0.0%)
**Prior MI (n = 52)**	14 (26.9%)	20 (38.5%)	10 (19.2%)	8 (15.4%)
**Prior PCI (n = 45)**	12 (26.6%)	18 (40.0%)	7 (15.5%)	8 (17.8%)
**Prior CABG (n = 10)**	2 (20.0%)	2 (20.0%)	2 (20.0%)	4 (40.0%)
Presenting symptoms				
**Typical angina chest pain (n = 94)**	12 (12.8%)	50 (53.2%)	20 (21.3%)	12 (12.8%)
**Atypical angina chest pain (n = 36)**	9 (25.0%)	17 (47.2%)	6 (16.7%)	4 (11.1%)
**No chest pain (n = 56)**	7 (12.5%)	30 (53.6%)	9 (16.1%)	10 (17.9%)
Killip class (preoperative)				
**1 (n = 81)**	12 (14.8%)	38 (46.9%)	20 (24.7%)	11 (13.6%)
**2 (n = 96)**	16 (16.7%)	54 (56.3%)	14 (14.6%)	12 (12.5%)
**3 (n = 12)**	1 (8.3%)	9 (83.3%)	0 (0.0%)	2 (16.7)
**4 (n = 1)**	0 (0.0%)	0 (0.0%)	0 (0.0%)	1 (100.0%)
MI localization in ECG				
**Anterior wall (n = 14)**	0 (0.0%)	8 (57.1%)	2 (14.3%)	4 (28.6%)
**Inferior wall (n = 10)**	2 (33.3%)	2 (33.3%)	0 (0.0%)	2 (33.3%)
**Anterolateral wall (n = 4)**	2 (50.0%)	0 (0.0%)	2 (50.0%)	0 (0.0%)

**Fig. 1:**
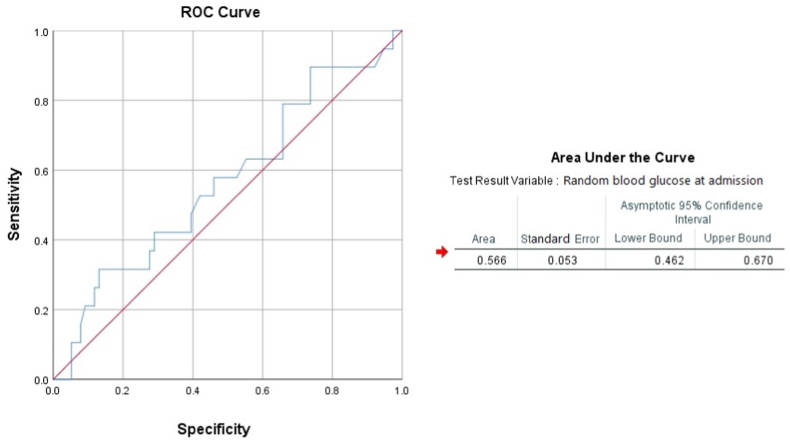
Receiver operating characteristic analysis for random blood glucose at admission predicting major adverse in-hospital events.

**Fig. 2:**
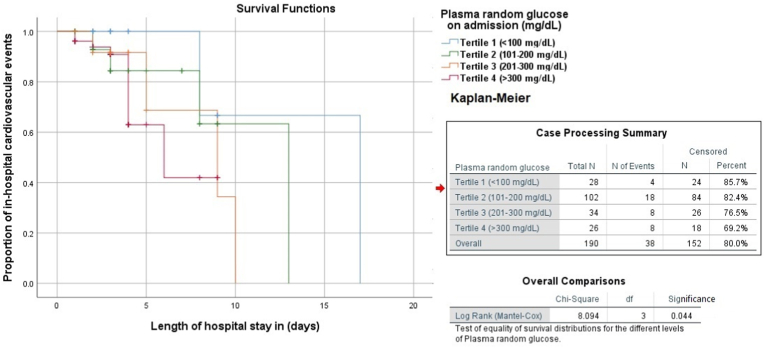
Kaplan-Meier log rank (Mantel-Cox) test signifying higher proportion of in-hospital events with higher tertiles of random blood glucose levels.

Both the incidence of all-cause deaths and cumulative rates of major adverse cardiovascular events were significantly the lowest in patients within tertile 1. The cumulative incidence of in-hospital events was 14.3% in tertile 1, 17.6% in tertile 2, 23.5% in tertile 3, and 30.8% in tertile 4. The odds ratio of major adverse cardiovascular events was 1.286 [0.397–4.161] in tertile 2, 1.846 [0.492–6.927] in tertile 3, and 2.667 [0.693–10.254] in tertile 4. Admission random glucose levels were significantly associated with a history of diabetes mellitus (DM), Killip class, inferior wall MI, three-vessel disease, recurrent MI, cardiogenic shock, and in-hospital mortality (Table 2). No significant differences were noted in other in-hospital events such as bleeding complications, severe arrhythmia, CHF treatment, and stroke (Table 3). Post-op TIMI score and average length of hospital stay were not significant either. Receiver operative characteristic curve on a cut-off 150.50 mg/dL plasma glucose level predicted adverse in-hospital event with a sensitivity of 57.9% and a specificity of 53.9% (AUC: 0.566 [0.462–0.670]). At cut-off 200.00 mg/dL, sensitivity becomes 42.1% and specificity increases to 71.1%, and at cut-off 261.50 mg/dL, specificity becomes 86.8% (Fig. 1). Kaplan-Meier curve shows that a higher cumulative proportion of in-hospital adverse events occurred with higher tertiles of random blood glucose levels (Log-rank test, p = 0.044) (Fig. 2).

**Table 2:** Association of angiographic profile of the study population.

Angiographic profile	Tertile 1 (<100 mg/dL)	Tertile 2 (101–200 mg/dL)	Tertile 3 (201–300 mg/dL)	Tertile 4 (>300 mg/dL)
**Infarct-related coronary artery**				
Left main coronary artery (n = 4)	2 (50.0%)	2 (50.0%)	0 (0.0%)	0 (0.0%)
Left anterior descending (n = 50)	6 (12.0%)	24 (48.0%)	11 (22.0%)	9 (18.0%)
Circumflex artery (n = 37)	4 (10.8%)	15 (40.5%)	6 (16.7%)	12 (32.4%)
Right coronary artery (n = 41)	8 (19.5%)	14 (34.1%)	11 (26.8%)	10 (21.9%)
**Severity of CAD**				
One-vessel (n = 26)	2 (7.7%)	14 (53.8%)	8 (30.8%)	2 (7.7%)
Two-vessel (n = 9)	0 (0.0%)	9 (100.0%)	0 (0.0%)	0 (0.0%)
Three-vessel (n = 43)	6 (13.9%)	12 (27.9%)	13 (30.2%)	12 (27.9%)
**Post-operative TIMI**				
3 (n = 112)	12 (10.7%)	62 (55.4%)	22 (19.6%)	16 (14.3%)
2 (n = 56)	10 (17.9%)	29 (51.8%)	7 (24.1%)	10 (17.9%)
0-1 (n = 22)	6 (27.3%)	12 (54.5%)	4 (18.2%)	0 (0.0%)
**Medical therapy**				
Aspirin (n = 156)	17 (10.3%)	89 (57.0%)	27 (17.3%)	23 (14.7%)
Clopidogrel/Tegreino (n = 94)	11 (11.7%)	51 (54.2%)	21 (22.3%)	11 (11.7%)
β-blockers (n = 127)	14 (11.0%)	70 (55.1%)	26 (20.5%)	17 (13.4%)
ACEI/ARBs (n = 102)	14 (13.7%)	56 (54.9%)	18 (17.6%)	14 (13.7%)
Statins@ (n = 144)	16 (11.1%)	82 (56.9%)	28 (19.4%)	18 (12.5%)
Successful PCI (n = 150)	22 (14.7%)	82 (54.7%)	28 (18.7%)	18 (12.0%)

**Table 3:** Association of major in-hospital outcomes with deranged blood glucose.

In-hospital outcomes	Tertile 1 (<100 mg/dL)	Tertile 2 (101–200 mg/dL)	Tertile 3 (201–300 mg/dL)	Tertile 4 (>300 mg/dL)
CHF treatment (n = 32)	2 (6.3%)	18 (56.3%)	8 (25.0%)	4 (12.5%)
Bleeding event (n = 4)	2 (50.0%)	0 (0.0%)	0 (0.0%)	2 (50.0%)
Severe arrhythmia (n = 3)	0 (0.0%)	1 (33.3%)	0 (0.0%)	2 (66.6%)
New-onset MI (n = 2)	0 (0.0%)	0 (0.0%)	0 (0.0%)	2 (100.0%)
Stroke (n = 1)	0 (0.0%)	0 (0.0%)	1 (100.0%)	0 (0.0%)
Cardiogenic shock (n = 2)	0 (0.0%)	0 (0.0%)	1 (50.0%)	1 (50.0%)
In-hospital death (n = 2)	0 (0.0%)	0 (0.0%)	0 (0.0%)	2 (100.0%)

## 4 Discussion

In the present study, we demonstrated the effects of impaired blood glucose levels in predicting in-hospital outcomes among patients with STEMI undergoing PCI. There are many studies performed previously, which analyzed the link between hyperglycemia and worse clinical outcomes in patients with STEMI. The common findings are that a large number of acute myocardial infarction (AMI) patients have altered glucose metabolism and overall hyperglycemia leads to increased mortality and morbidity [7,8]. One retrospective study conducted in Switzerland having 1288 MI events with a 15-month follow-up duration showed that patients with raised admission blood glucose levels undergoing PCI have a reduced survival rate [8]. Similarly, our findings pointed out that a higher glycemic index increases the probability of in-hospital mortality in STEMI among post-PCI patients. Since all the patients included in our analysis were not diabetic, there is a high possibility of incident hyperglycemia with stress and acute myocardial event.

In STEMI patients who are not known to have diabetes, hyperglycemia at the time of admission indicates intense cardiac dysfunction [9]. Interestingly, it is suggested that the raised plasma glucose levels in non-diabetics might be due to advanced ischemia or hemodynamic damage [9]. There are certain stress mediators which are released during myocardial infarction that cause high blood glucose levels independent of insulin secretion [8]. Moreover, in general, hyperglycemia negatively impacts endothelium-dependent vasodilation and functionality of macrophages and lymphocytes [10]. Besides that, raised blood glucose levels also increase osmotic diuresis which causes a decline in circulating, end-diastolic and stroke volumes by interfering with Frank-Starling mechanism of myocardial contractility [10]. But still, diabetes remains a strong predisposing factor for cardiovascular complications such as atherosclerosis including both large arteries and coronary arteries [4]. The study conducted by Ingo Eitel et al. [3] on 411 MI patients with an average follow-up period of 18–19 months found that pre-existing diabetes remains a significant predictor of MACE, similar to findings of the current study. DM patients with poor glycemic control or stress hyperglycemia on admission experienced highest rates of MACE including death.

Previously many studies attempted to link hyperglycemia with altered TIMI flow during PCI. It is supposed that hyperglycemia leads to raised oxidative stress, adhesion molecules, and tissue factor as well as potentiates thrombin formation, platelet activation, and fibrinolytic resistance [11]. This sets a prothrombotic environment which results in no flow phenomena, ultimately leading to worse TIMI flow after primary PCI [11]. But this association remains unclear. A study conducted in Indonesia by a group of researchers showed that hyperglycemic STEMI patients had worse final TIMI flow after primary PCI [11]. In contrast, our study showed that post-op TIMI score was not significant after PCI in patients who had hyperglycemia at the time of admission.

When it comes to in-hospital complications such as bleeding, severe arrhythmias, stroke, and congestive heart failure (CHF), there were no significant differences noted in STEMI patients with hyperglycemia on admission. One case-control study was done in Poland on 529 patients with short-term (in-hospital) and 1-year (long-term) outcomes also observed that there was no association between raised blood glucose levels on admission with a higher risk of bleeding and ischemic stroke [12].

A case-control study conducted in Russia also predicted the outcomes of STEMI patients based on blood glucose measurements at admission to hospital [13]. Similarly, they found an association between deranged blood glucose levels and in-hospital mortality on 958 retrospectively enrolled both diabetic and non-diabetic patients. Moreover, the study also demonstrated a one-year prognosis for overall mortality and cardiovascular events [13]. Qin and colleagues from China also studied the relationship of random and fasting blood glucose levels with severity of same 958 acute MI patients and found fasting blood glucose more independently predicted risk factor among them [14]. While predicting in-hospital mortality, they reported an AUC of 0.789 for random blood glucose and 0.810 for fasting blood glucose, both of which were significantly higher than our study [15]. Despite the American Diabetes Association recommendations of keeping the therapeutic target of glucose control between 140 and 180 mg/dL during hospital stay and intensive care [16], there have been studies suggesting higher mortality rates with each 10-mg/dL rise in mean glucose levels [17]. Wahab et al. have calculated the odds for in-hospital mortality in AMI patients of 2.44 [1.42–4.20] in non-diabetic and 1.91 [1.16–3.14] with random blood glucose >198 mg/dL, which are comparable with our findings [18].

The mechanisms by which glucose management in ACS may improve outcomes in hyperglycemic patients include a decline in inflammatory and clotting mediators, reduced infarct size, and improvement of endothelial function and fibrinolysis [19]. On the contrary, spontaneous hypoglycemia is also happened to be a risk factor for mortality following MI [20]. While some studies also reported short and long-term outcomes of AMI by using glycemic gap [21,22]. There is also uncertainty over diagnosing incident diabetes during acute myocardial event among previously undiagnosed cases [23]. But mortality is mostly reported higher in non-diabetic patients with hyperglycemia as compared to known diabetics at the time of acute cardiac event [24]. A meta-analysis conducted on AMI patients showed a high prevalence of new-onset glucose intolerance among those with MACE and mortality [25].

There were some potential limitations of the current study not only pertaining to limited sample size and single-center investigation. We did not take into account long-term follow-up outcomes and complications since the study was focused only on the in-hospital stay outcomes following PCI. Also, single random blood glucose reading was used to determine the association of hyperglycemia with severe events, however, serial measurements should be done in order to determine the glycemic control over time. Further, in-hospital management and use of basal insulin were not taken into account while reporting the blood sugar levels. We also did not discriminate among the diabetic and non-diabetic population in our cohort, while focusing on the deranged blood glucose levels. Lastly, HbA1c would have been an ideal indicator of poor glycemic control in this population, but was not available in all the cases and hence could not be analyzed.

## 5 Conclusion

Strict control of the glycemic index may improve the survival of patients who have both DM and coronary artery disease. DM patients with poor glycemic control or stress hyperglycemia on admission experienced the highest rates of major adverse cardiovascular events including deaths. Plasma random glucose was predictive of a worse prognosis for STEMI patients undergoing PCI in our study.

## Conflict of interest disclosure

The authors have no conflict of interest.

## Registration of research studies

1. Name of the registry: Dow University Hospital.

2. Unique Identifying number or registration ID: IRB/DUH/2020/784.

3. Hyperlink to your specific registration (must be publicly accessible and will be checked):

## Guarantor

Muhammad Sohaib Asghar.

## Funding statement

The authors declare that they have no commercial associations (e.g. consultancies, stock ownership, equity interest, patent/licensing arrangement etc.) or funding with this article.

## Data availability statement

Data can be made available on request from corresponding author.

## Ethical approval statement

Ethical approval was taken in this study from institutional review board of Dow University Hospital (Ref:App.# IRB/DUH/2020/784).

## Patient consent

Consent to participate from the patients was waived and not required due to retrospective nature of the data collection.

## Provenance and peer review

Not commissioned, externally peer reviewed.

## Author contribution

T.A.K and M.S.A conceived the idea; M.S, S.K, K.K, M.S.A, O.P and S, collected the data; T.A.K, F.Y and M.S.A analyzed and interpreted the data; N.C, F.K, and T.A.K did write up of the manuscript; and finally, M.S.A and F.Y reviewed and revised the manuscript for intellectual content critically. All authors approved the final version of the manuscript.
